# Comparative efficacy and safety of non-pharmacological interventions on treatment-induced xerostomia in head and neck cancer patients: a systematic review and network meta-analysis

**DOI:** 10.3389/fonc.2025.1644178

**Published:** 2025-07-30

**Authors:** Jingyu Tai, Aimin Guo, Juntong Chen, Haoyu Li, Qian Dong, Wumei Hao, Wenjing Wang, Zunzhu Li, Jianshu Ye, Jinbang Liu, Chengwu Yang

**Affiliations:** ^1^ School of Nursing, Peking Union Medical College, Beijing, China; ^2^ Intensive Care Medicine Department of Peking Union Medical College Hospital, Beijing, China

**Keywords:** head and neck cancer, Xerostomia, non-pharmacological intervention, network meta-analysis, evidence-based

## Abstract

**Aim:**

To comprehensively evaluate the efficacy and safety of non-pharmacological interventions for radiotherapy-induced xerostomia in patients with head and neck cancer.

**Methods:**

A systematic literature search was conducted in PubMed, Embase, the Cochrane Library, and Web of Science for articles published up to March 1, 2025. Three outcome measures were utilized to assess treatment effectiveness: xerostomia, saliva flow rate, and xerostomia-related quality of life. A Bayesian network meta-analysis was employed to synthesize the comparative performance of different non-pharmacological interventions. The study protocol was registered in the PROSPERO database (CRD420251027019).

**Results:**

A total of 30 RCTs encompassing 1,595 participants and nine distinct non-pharmacological treatment modalities were included. Compared with the SOC, mouthwash demonstrated the most pronounced improvement in XQ (SMD = -0.70; 95% CI: -1.38 to -0.01) and XI scores (SMD = -0.68; 95% CI: -1.09 to -0.26). Oral moisturizing gel exhibited the greatest reduction in VAS scores (SMD = -1.55; 95% CI: -2.31 to -0.80). Regarding salivary flow enhancement, oral moisturizing gel was most effective in increasing USFR (SMD = 3.83; 95% CI: 0.56 to 7.09), while chewing gum provided the highest gain in SSFR (SMD=3.66; 95%CI: -0.08 to 7.41). Among safety outcomes, electrical stimulation therapy was associated with the most favorable profile relative to SOC (SMD = -1.82; 95% CI: -3.96 to 0.33).

**Conclusions:**

Non-pharmacological interventions appear to offer superior efficacy with comparable safety to SOC in care of radiotherapy-induced xerostomia among patients with head and neck cancer. Mouthwash is likely the most effective option for alleviating subjective xerostomia symptoms, with oral moisturizing gel as a valuable alternative. For salivary flow enhancement, oral moisturizing gel is preferred for unstimulated flow, whereas chewing gum is optimal for stimulated flow. Electrical stimulation therapy may yield the most substantial improvement in quality of life, with photobiomodulation therapy representing a promising adjunctive strategy.

**Systematic review registration:**

Prospective Register of Systematic Reviews (PROSPERO), identifier CRD420251027019.

## 1 Introduction

Head and neck cancer (HNC) ranks as the sixth most prevalent malignancy globally, with over 891,000 new cases reported annually (1, 2). Radiotherapy (RT), administered either alone or in combination with surgery or chemotherapy, remains a cornerstone of curative treatment for HNC. Approximately 40% of patients with early-stage disease receive definitive RT (3). Among the most prevalent adverse effects of RT are oral mucositis (OM) and xerostomia, with up to 80% of patients experiencing some degree of dry mouth during cancer therapy (4, 5). Xerostomia commonly manifests within the initial weeks of therapy and may persist well beyond its conclusion, in some cases lasting up to two years (6). Reduced or absent salivary secretion causes substantial discomfort and can significantly impair speaking, mastication, swallowing, and sleep. Additional complications include dysgeusia, inadequate nutritional intake, weight loss, dental caries, laryngopharyngeal reflux, and nocturia (7, 8). Pharmacologic management typically involves muscarinic receptor agonists such as pilocarpine and cevimeline, which stimulate residual salivary gland function. While these agents are considered first-line therapy, their utility is often constrained by cholinergic side effects, including diaphoresis, urinary frequency, flushing, chills, rhinitis, nausea, diarrhea, and potentially serious cardiovascular events such as bradycardia and hypotension (9–12). Conventional supportive measures, including frequent water sipping or sucking on ice chips, may offer transient relief through oral hydration. However, excessive fluid intake can dilute mucosal secretions and paradoxically worsen xerostomia symptoms (13). Moreover, increased nighttime fluid consumption may lead to nocturia and disrupt sleep continuity (10). These limitations have underscored the need for alternative therapeutic strategies that deliver effective symptom control while minimizing adverse events.

Over the past decade, an expanding body of evidence has highlighted the potential of non-pharmacological interventions in mitigating radiation-induced xerostomia among HNC patients. With the advent of numerous high-quality randomized controlled trials (RCTs), various treatment modalities have emerged. Despite these advances, which intervention achieves the most favorable balance between efficacy and safety remains uncertain. Topical mucosal lubricants and saliva substitutes, such as animal-derived mucins, carboxymethyl cellulose (CMC), and xanthan gum—have demonstrated symptomatic benefits in this context (14). These agents are typically formulated as moisturizing gels, sprays, toothpastes, or mouthwashes, but patient adherence is often influenced by preferences in texture, taste, and ease of use, which may ultimately affect clinical outcomes (15). Mechanical and gustatory stimulants, such as chewing gum or malic acid lozenges, are often employed as adjunctive oral care measures to alleviate discomfort and reduce the risk of complications associated with hyposalivation (16). Chewing gum, in particular, has been shown to increase salivary output in individuals with residual gland function, enhance oral pH, and improve buffering capacity (17). Acupuncture has also been reported to stimulate salivary secretion by modulating parasympathetic and sympathetic nervous systems, thereby improving long-term quality of life in affected individuals (18, 19). Similarly, transcutaneous electrical nerve stimulation (TENS) of the salivary glands—either through direct application or acupuncture-like methods—has shown potential in enhancing glandular function and increasing salivary output (16–20). Hyperbaric oxygen therapy (HBOT), which fosters angiogenesis and stem cell mobilization, has also emerged as a promising modality for restoring irradiated gland function (21).

Given that most RCTs have compared non-pharmacological interventions against standard care, rather than directly comparing different interventions with each other, there is a lack of head-to-head evidence regarding the relative efficacy of these strategies. To address this gap, the present study employed a Bayesian network meta-analysis framework to indirectly compare the effectiveness and safety of various non-pharmacological treatments for radiation-induced xerostomia in HNC patients. By synthesizing direct and indirect evidence, this analysis aimed to identify the most effective first-line and second-line therapy strategies tailored to different patient subgroups. The findings are expected to provide robust evidence-based guidance for optimizing clinical nursing practices in this population.

## 2 Materials and methods

This network meta-analysis (NMA) was conducted in accordance with the Preferred Reporting Items for Systematic Reviews and Meta-Analyses extension statement for network meta-analyses (PRISMA-NMA) (**Supplementary Table 1**) (22). Given the limited availability of head-to-head randomized controlled trials (RCTs) comparing different strategies for xerostomia management, a Bayesian framework was adopted to facilitate indirect comparisons and probabilistic ranking of intervention efficacy (23). To ensure methodological transparency and reproducibility, the study protocol was prospectively registered with the International Prospective Register of Systematic Reviews (PROSPERO), registration number CRD420251027019.

### 2.1 Data sources and search strategy

A systematic literature search was conducted in four major databases: PubMed, EMBASE, the Cochrane Library, and Web of Science. The search strategy combined both free-text terms and controlled vocabulary (MeSH/Emtree) and included the following terms: “Xerostomia,” “Asialia,” “Mouth Dryness,” “Hyposalivation,” “Thirst,” “head and neck neoplasms,” “randomized clinical trial,” “cold temperature,” “Low-Level Light Therapy,” “Chewing Gum,” “Menthol,” “Acupuncture,” “Electric Stimulation,” “ear acupressure,” and “Psychosocial Intervention.” No language restrictions were applied, and the search covered all records from database inception through March 1, 2025. Details of the complete search strategy are provided in **Supplementary Figure 1**.

### 2.2 Study selection criteria

Inclusion Criteria:

(1) RCTs involving patients diagnosed with HNC who developed xerostomia and/or salivary gland hypofunction of any etiology following radiotherapy.
(2) Studies evaluating one or more non-pharmacological interventions (either alone or in combination) aimed at managing xerostomia.
(3) Comparisons between non-pharmacological strategies and other interventions or standard care.
(4) Studies reporting at least one of the following outcomes: Xerostomia Questionnaire (XQ), Xerostomia Inventory (XI), Visual Analogue Scale (VAS), Unstimulated Salivary Flow Rate (USFR), Stimulated Salivary Flow Rate (SSFR), or Xerostomia-related Quality of Life. The XQ is a validated instrument for assessing xerostomia severity, with scores scaled from 0 to 100; a 10-point difference is considered clinically meaningful (24). The XI consists of 11 items rated on a 5-point Likert scale, yielding a total score ranging from 11 to 55, where higher scores indicate greater symptom burden (25). The VAS quantifies perceived dry mouth severity on a 0–10 scale (26). USFR and SSFR assess baseline and stimulated salivary output, respectively—stimuli for SSFR typically include chewing or acid exposure (19). The XeQoL consists of 14 items spanning physical, discomfort, psychological, and social domains, with scores ranging from 0 to 60 (higher scores reflecting worse quality of life).

Exclusion Criteria:

(1) RCTs reporting on different phases of the same patient cohort.
(2) Studies with unclear or insufficient outcome reporting.
(3) Non-original studies, including reviews, case reports, and editorials.

Initial screening was performed based on titles and abstracts. Two reviewers conducted Full-text assessments independently to determine final eligibility, with discrepancies resolved by consensus.

### 2.3 Data extraction and risk of bias assessment

Data extraction was performed independently by three reviewers using a standardized form, with discrepancies adjudicated by a fourth reviewer. Extracted data included authorship, year of publication, sample size, patient demographics, intervention details, comparator information, and all relevant outcome measures (XQ, XI, VAS, USFR, SSFR, XeQoL). Methodological quality of the included studies was appraised using the Cochrane Risk of Bias Tool (RoB 2.0) (12), which evaluates five domains: (1) Bias arising from the randomization process; (2) Bias due to deviations from intended interventions; (3) Bias due to missing outcome data; (4) Bias in outcome measurement; (4) Bias in selection of the reported result. Each domain was rated as low risk, high risk, or “some concerns”.

### 2.4 Statistical analysis

Reference management was performed using EndNote X9, and data organization was conducted in Microsoft Excel 2021. Bayesian network meta-analyses were executed using Stata version 17.0. For dichotomous variables, odds ratios (OR) with 95% credible intervals (CrI) were used; for continuous outcomes, either mean differences (MD) or standardized mean differences (SMD) were applied, depending on the consistency of measurement scales across studies.

A network evidence map was constructed, where node size represented the number of participants per intervention, and edge thickness corresponded to the number of direct comparisons. For open loops in the network, a consistency model was applied. For closed loops, inconsistency was assessed using loop-specific tests; a P-value > 0.05 indicated acceptable consistency between direct and indirect estimates. In the presence of significant inconsistency, subgroup analyses and meta-regression were performed to explore potential sources of heterogeneity. Treatment efficacy rankings were estimated using the surface under the cumulative ranking curve (SUCRA), with values ranging from 0 to 100 higher values indicating greater efficacy. Inconsistency factors (IFs) were also calculated for closed loops, with consistency considered acceptable if the 95% CrI of the IF included zero. Finally, comparison-adjusted funnel plots were generated to assess potential small-study effects and publication bias.

## 3 Results

### 3.1 Systematic review and characteristics of included studies

**Figure 1:**
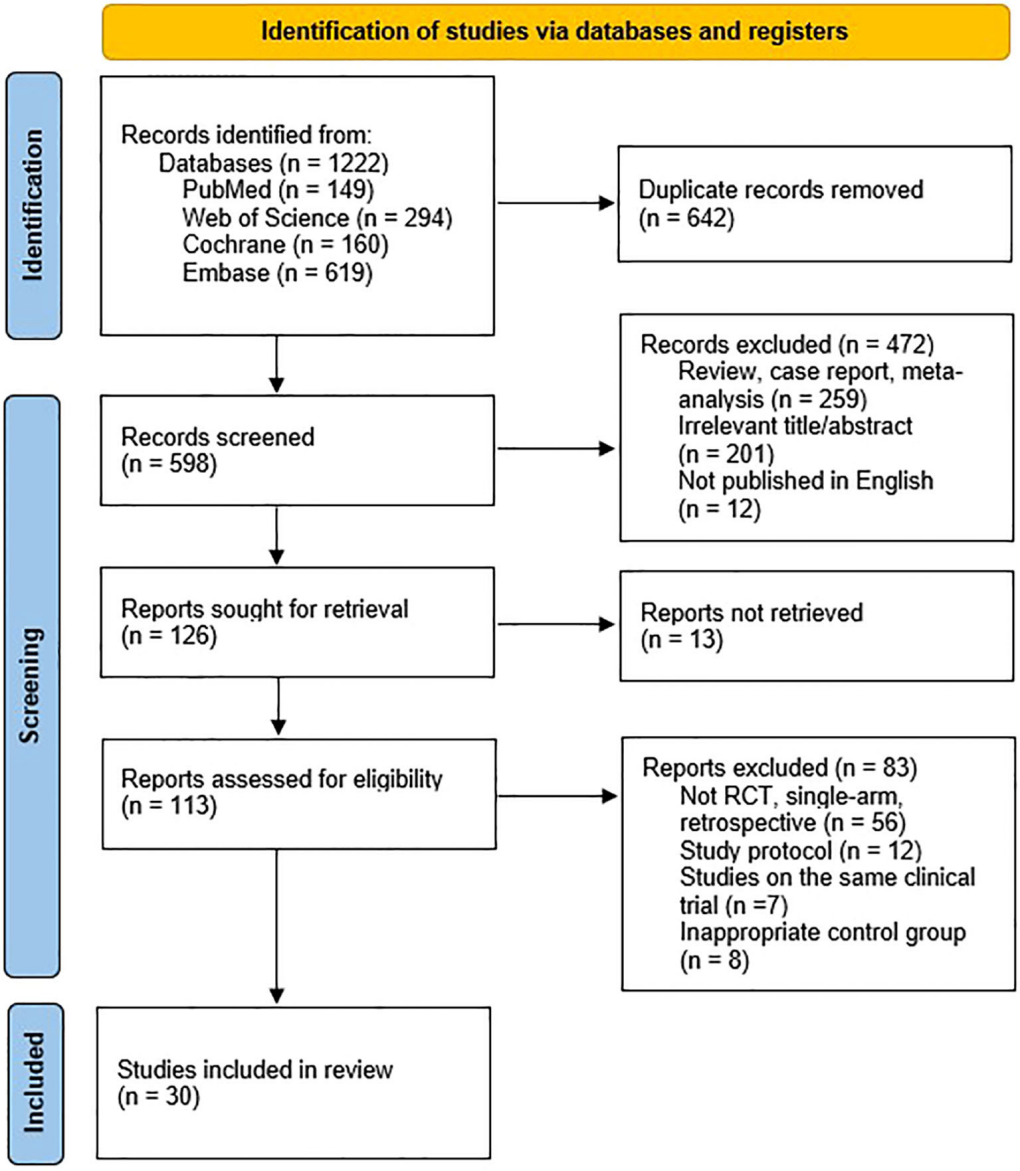
Literature search and study selection flowchart, conducted in accordance with the PRISMA guidelines.

An initial search across databases yielded 1,222 records. After removing duplicates and screening titles and abstracts for relevance, 598 studies were selected for full-text evaluation (**Figure 1**). Ultimately, 30 studies (19, 27–55) met the pre-specified inclusion criteria, encompassing a total of 1,595 patients who underwent one of nine non-pharmacological interventions: acupuncture, photobiomodulation or laser therapy (LT), chewing gum, transcutaneous electrical nerve stimulation (TENS), oral spray, mouthwash, oral balance gel (GEL), supersaturated humidification therapy (STHT), or hyperbaric oxygen therapy (HBOT). In most trials, the standard of care (SOC) was the control. A detailed summary of study characteristics is provided in **Table 1**.

**Table 1:** Baseline characteristics of included studies.

Authors year	Ethnicity	Age, years mean ± SD	Sample	Male/Female	Intervention arm(s)	Control arm	Outcome measures
Cohen et al., 2024 (19)	American	64.4 ± 9.9 65.6 ± 9.0	86/86	201/57	Acupuncture	Standard of care	XeQoL, XQ
Silva et al., 2023 (44)	American	59 ± 39.0 62 ± 35.8	26/27	42/11	Photobiomodulation	Standard of care	XeQoL
kaae et al., 2020 (27)	Europe	61 60.5	55/36	84/54	Gum	Standard of care	SSFR, XeQoL
Mozaffari et al., 2024 (38)	Asian	54.1 ± 15.4 57 ± 15.18	17/20	26/11	Photobiomodulation	Standard of care	SSFR
Iovoli et al., 2020 (54)	American	61.1 ± 7.5 62.6 ± 8.9	15/15	26/4	Electrical Nerve Stimulation	Standard of care	XeQoL
Louzeiro et al., 2020 (33)	North America	/	10/11	/	photobiomodulation	Standard of care	XeQoL, VAS, USFR, SSFR
Mercadante et al., 2024 (48)	Europe	58.4 ± 10.8 58.2 ± 9.3	36/32	66/20	Salivary Electrostimulation	Standard of care	XeQoL, VAS, SSFR
Piboonratanakit et al., 2023 (53)	Asian	54.1 ± 13.9 58.3 ± 14.8	35/35	44/25	Trehalose Oral Spray	Standard of care	XeQoL, VAS, USFR
Marimuthu et al., 2021 (47)	Asian	/	47/47	63/31	Saliva substitute mouthwash	Standard of care	XI, USFR
Meng Z et al., 2012 (46)	Asian	45.6 ± 10.8 48.9 ± 10.5	29/33	59/25	Acupuncture	Standard of care	XQ, USFR, SSFR
Blom et al., 1996 (29)	Europe	60 ± 9.7 63.72 ± 8.4	20/18	26/12	Acupuncture	Standard of care	USFR, SSFR
Carcia et al., 2019 (34)	American	46.3 ± 11.5 46.4 ± 10.4	46/37	70/17	Acupuncture	Standard of care	XQ
Cho et al., 2008 (43)	Europe	49.2 ± 10.6 48.7 ± 31.4	6/6	/	Acupuncture	Standard of care	XQ, USFR, SSFR
Pfister et al., 2010 (28)	American	61 57	28/30	38/20	Acupuncture	Standard of care	XI
Agna et al., 2021 (50)	South America	62.7 ± 11.1 61.5 ± 10.6	47/52	90/17	Acupuncture	Standard of care	XI
Lakshman et al., 2015 (36)	Asian	/	10/10	/	Salivary Electrostimulation	Standard of care	USFR, SSFR
Paim et al., 2019 (35)	South America	59.9 ± 5.8 57.5 ± 8.1	37/31	63/4	Salivary Electrostimulation	Standard of care	SSFR
Saleh et al., 2024 (32)	South America	58.7 ± 9.1 55.6 ± 8.7	12/11	15/8	Low-Level Laser Therapy	Standard of care	USFR, SSFR, XeQoL
Gonnelli et al., 2016 (41)	South America	/	13/10	20/3	Low-level laser therapy	Standard of care	USFR, SSFR
Barbosa et al., 2018 (37)	South America	40.5 ± 12.5 61.0 ± 27.6	15/29	36/8	Low-level laser therapy	Standard of care	VAS, SSFR
Fidelix et al., 2018 (42)	South America	53.9 ± 11.7 57.2 ± 11.59	33/33	2/64	Low-level laser therapy	Standard of care	XI, SSFR
Sugaya et al., 2016 (40)	South America	57.3 ± 49.7 62.7 ± 22.6	13/10	2/21	Low Intensity laser therapy	Standard of care	VAS
Nagy et al., 2007 (55)	Europe	57.9 ± 10.5 58 ± 9.3	18/18	20/16	Biotène Oral Balance gel	Standard of care	VAS, USFR
Criswell et al., 2001 (39)	American	/	12/12	20/4	Supersaturated humidification	Standard of care	VAS
Momm et al., 2005 (31)	Europe	/	60/60	89/31	Oral Balance gel	Mucin spray	XI, USFR
Paterson et al., 2019 (45)	Europe	62 ± 30 58 ± 25.6	25/14	35/4	Visco-ease™ oral spray	Standard of care	XI
Cankar et al., 2011 (51)	Europe	56.3 ± 1.9	8/8	9/7	Hyperbaric Oxygenation	Standard of care	USFR
Charalambous et al., 2017 (52)	American	59.9 ± 12.7 63.1 ± 14.3	36/36	52/20	Thyme honey mouthwash	Standard of care	XQ, XeQoL
Cosimo et al., 2023 (49)	Europe	54.2 ± 10.4 55.1 ± 10.8	16/16	19/13	Sodium-hyaluronate mouthwash	Standard of care	XQ
Aagaard et al., 1992 (30)	Europe	/	43/39	/	Gum	Standard of care	USFR, SSFR

The risk of bias was assessed using the Cochrane Risk of Bias 2.0 (ROB 2.0) tool. Among the included studies, 15 were rated as having an overall low risk of bias, while the remaining 15 were considered to have some concerns. Notably, two studies (30, 55) did not employ randomization. Six studies (30, 36, 37, 44, 47, 53) were flagged as having “some concerns” related to deviations from intended interventions, while eight (27, 34, 35, 39, 46, 49, 50, 55) were rated as “high risk” in the same domain. No studies exhibited high risk in missing outcome data, outcome measurement, or selective reporting domains. A comprehensive summary is provided in **Supplementary Figure 2**.

### 3.2 Network meta-analyses

#### 3.2.1 Primary outcome: xerostomia

##### 3.2.1.1 XQ scores

**Figure 2:**
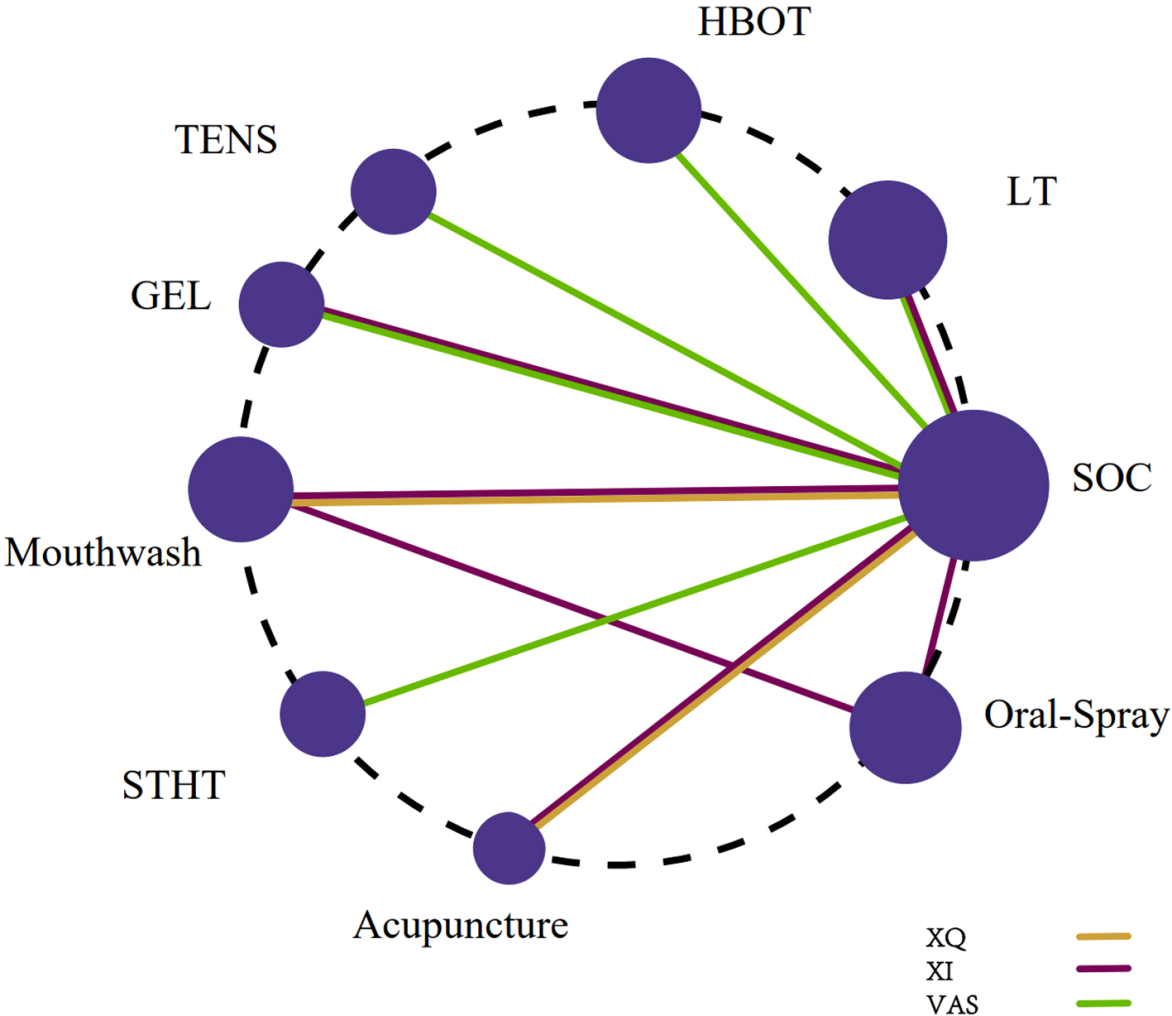
Network plot of non-pharmacological interventions for xerostomia in patients with HNC following radiotherapy, based on efficacy outcomes in XQ, XI, and VAS scores.

Six studies (19, 34, 43, 46, 49, 52) utilized the XQ to assess xerostomia. The network meta-analysis incorporated three interventions: Acupuncture, Mouthwash, and SOC. The treatment network is depicted in **Figure 2**.

**Figure 3:**
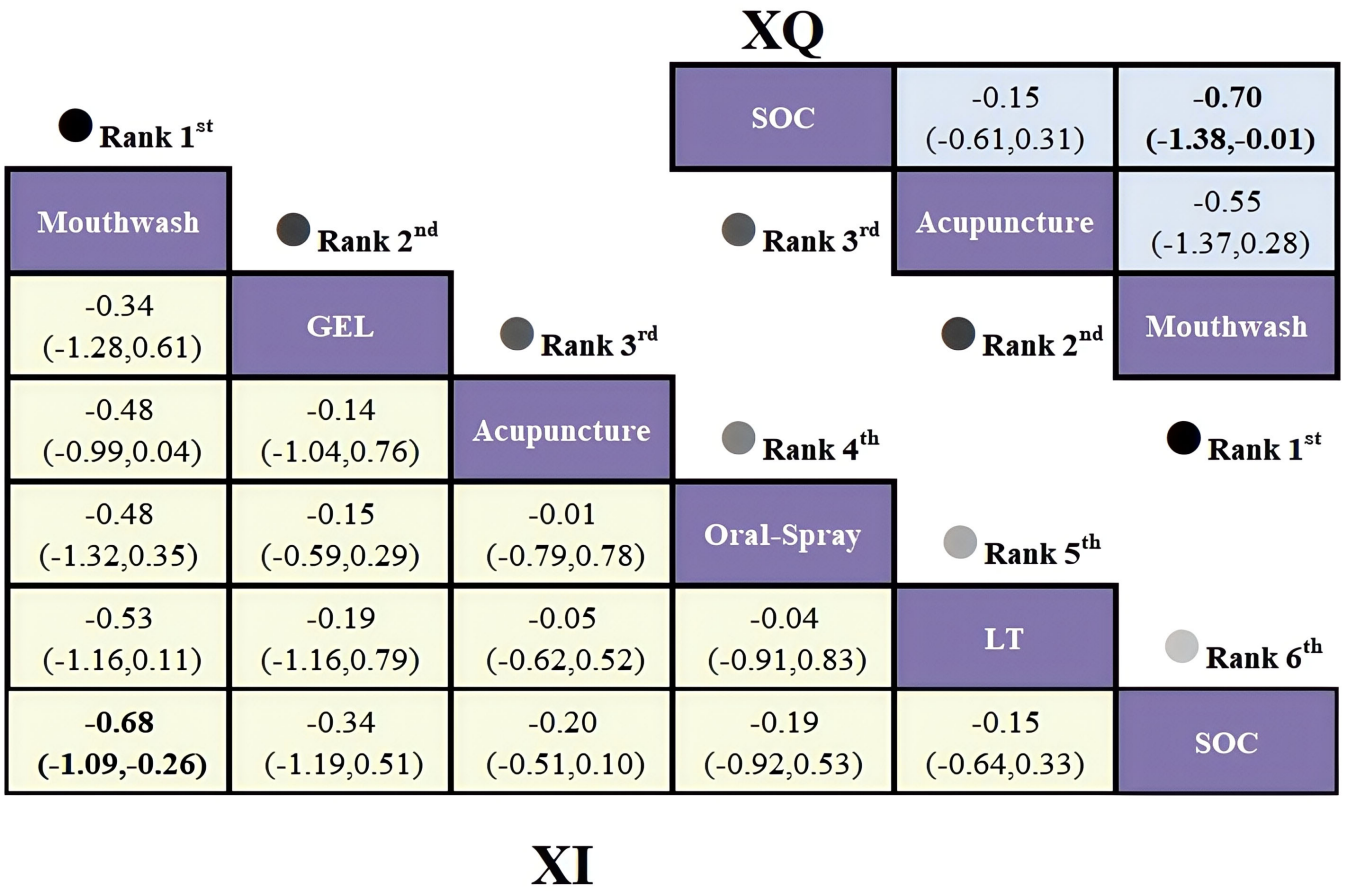
League table based on Bayesian network meta-analysis comparing the efficacy and safety of non-pharmacological interventions for xerostomia in patients with HNC following radiotherapy. A statistically significant difference is indicated when the SMD values and their corresponding 95% confidence intervals for both XQ and XI are either entirely above or below zero.

Given the open-loop network structure, a consistency model was applied. Analysis of standardized mean differences (SMDs) and 95% confidence intervals (CIs) revealed that mouthwash provided the greatest relief from xerostomia compared with SOC (SMD = -0.70; 95% CI [-1.38, -0.01]), with a statistically significant difference. Acupuncture also showed favorable effects versus SOC, though not statistically significant. Results are presented in **Figure 3**.

**Figure 4:**
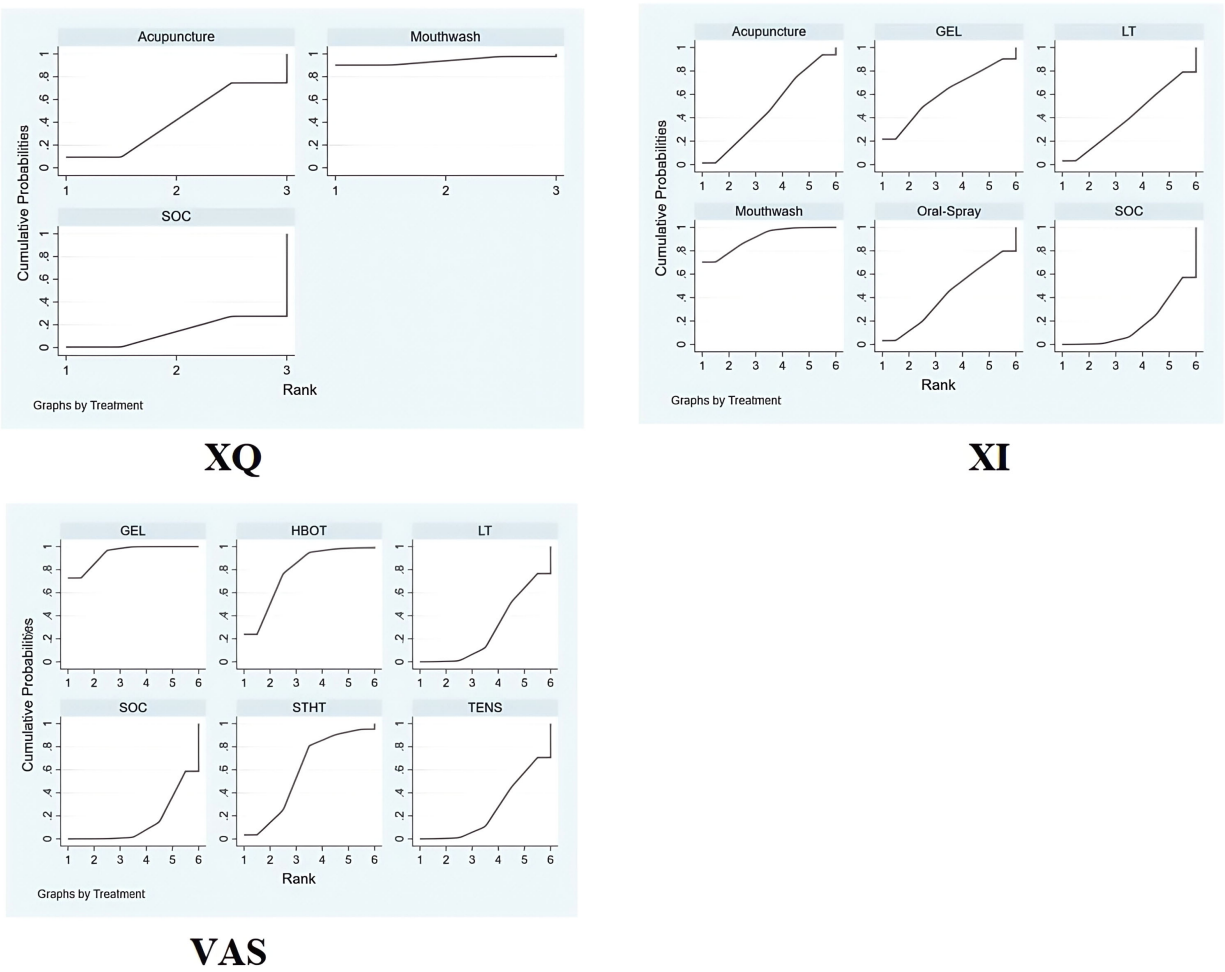
Bayesian ranking plot of non-pharmacological interventions for xerostomia in patients with HNC following radiotherapy, based on efficacy outcomes in XQ, XI, and VAS scores. 95% CI [-2.21, -0.12]), with a statistically significant difference.

The SUCRA-based ranking indicated that Mouthwash was most effective (94.0%), followed by Acupuncture (42.0%) and SOC (14.0%) (**Figure 4**).

##### 3.2.1.2 XI scores

Six studies (28, 31, 42, 45, 47, 50) employed the XI. This network included six interventions: Acupuncture, Mouthwash, Oral Moisturizing Gel, Oral Spray, Photobiomodulation, and SOC (**Figure 2**).

The network meta-analysis based on the XI revealed an open-loop structure; therefore, a consistency model was applied. The analysis of standardized mean differences (SMDs) and their 95% confidence intervals indicated that, compared to routine care, mouthwash demonstrated the most significant efficacy in alleviating xerostomia in patients with HNC (SMD = -0.68, 95% CI [-1.09, -0.26]), with a statistically significant between-group difference. Acupuncture and oral spray yielded comparable effects (SMD = -0.01, 95% CI [-0.79, 0.78]), while oral spray and photobiomodulation therapy showed similar efficacy (SMD = -0.04, 95% CI [-0.91, 0.83]); however, none of these differences reached statistical significance. Detailed results are presented in **Figure 3**.

Rankings based on SUCRA were: Mouthwash (90.7%) > Oral Gel (60.9%) > Acupuncture (47.7%) > Oral Spray (42.3%) > Photobiomodulation (40.5%) > SOC (17.8%) (**Figure 4**).

##### 3.2.1.3 VAS scores

Seven studies (32, 37, 39, 40, 48, 51, 55) utilized the Visual Analogue Scale (VAS). The network encompassed six interventions: Oral Moisturizing Gel, Photobiomodulation, HBOT, STHT, TENS, and SOC (**Figure 2**).

The network based on the VAS outcome revealed an open-loop structure; thus, a consistency model was applied for analysis. Results of the standardized mean differences (SMDs) and their 95% confidence intervals indicated that oral moisturizing gel was the most effective intervention for relieving xerostomia in HNC patients, showing significantly greater efficacy than routine care (SMD = -1.55, 95% CI [-2.31, -0.80]), as well as compared to photobiomodulation therapy (SMD = -1.43, 95% CI [-2.32, -0.54]) and TENS (SMD = -1.47, 95% CI [-2.36, -0.58]), all with statistically significant between-group differences. In addition, HBOT also demonstrated superior efficacy over routine care (SMD = -1.12, 95% CI [-2.21, -0.12]), with a statistically significant difference. Detailed results are provided in **Figure 8**.

Intervention ranking based on SUCRA: Oral Gel (93.9%) > HBOT (78.5%) > STHT (59.0%) > Photobiomodulation (28.3%) > TENS (25.4%) (**Figure 4**).

#### 3.2.2 Primary Outcome: Saliva flow rate

##### 3.2.2.1 USFR

**Figure 5:**
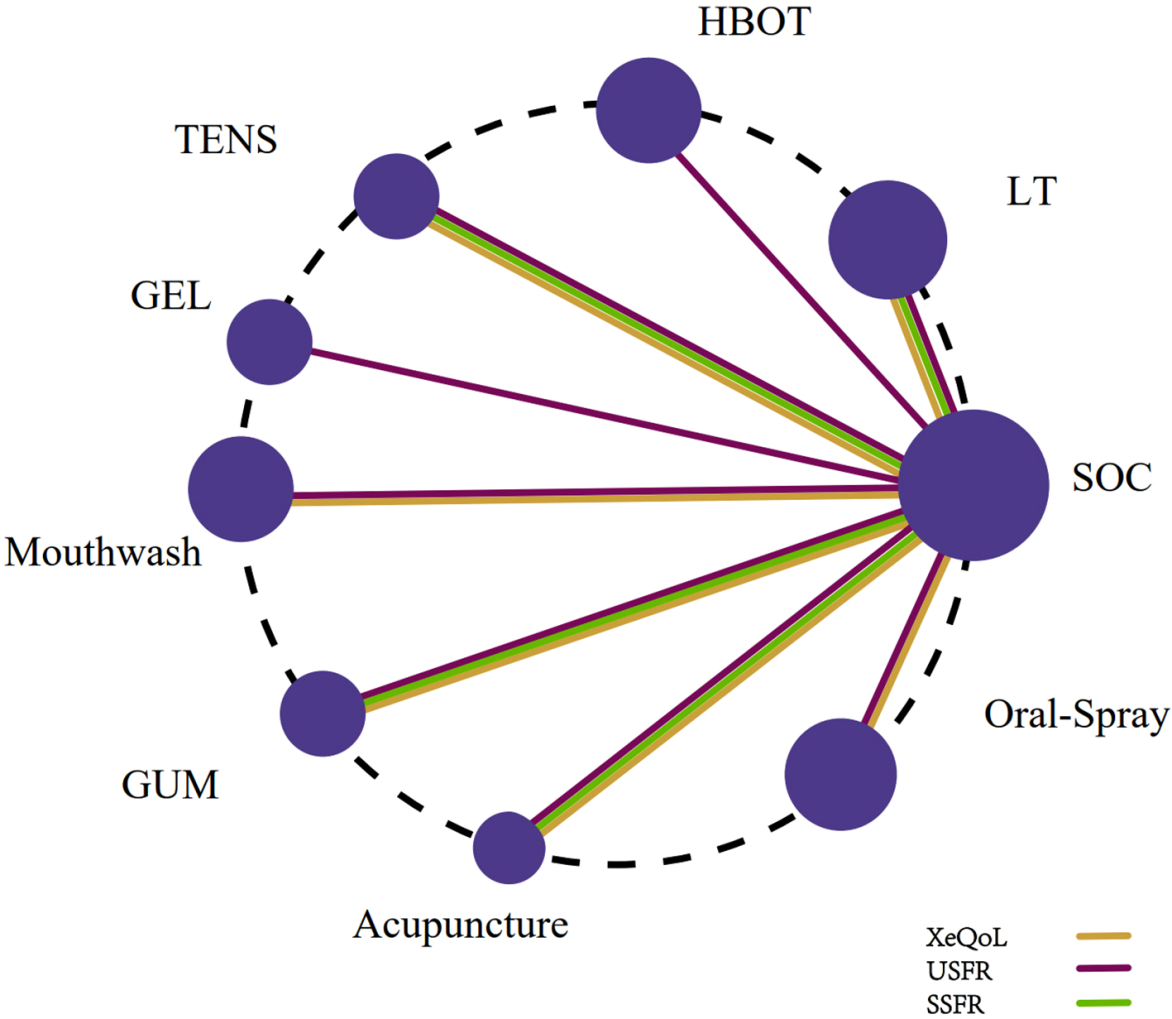
Network plot of non-pharmacological interventions for xerostomia in patients with HNC following radiotherapy, based on efficacy outcomes in USFR, SSFR, and XeQoL scores.

Fifteen studies (27–30, 32, 36, 37, 41, 43, 46–48, 51, 53, 55) reported USFR as the outcome. The network included nine interventions: Oral Gel, Photobiomodulation, Chewing Gum, HBOT, Oral Spray, TENS, Mouthwash, Acupuncture, and SOC (**Figure 5**).

**Figure 6:**
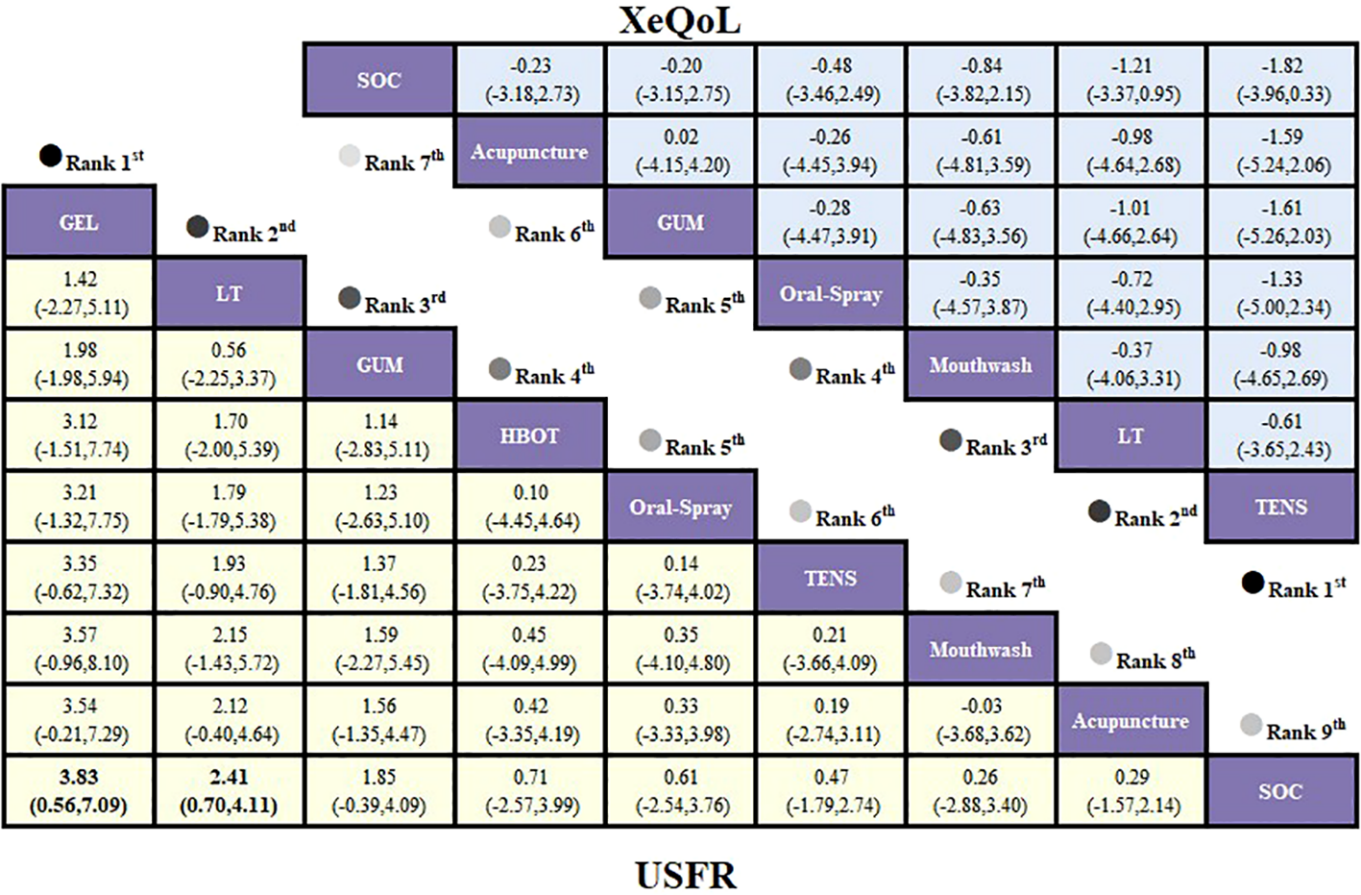
League table based on Bayesian network meta-analysis comparing the efficacy and safety of non-pharmacological interventions for xerostomia in patients with HNC following radiotherapy. A statistically significant difference is indicated when the SMD values and their corresponding 95% confidence intervals for both USFR and XeQoL are either entirely above or below zero.

The network meta-analysis based on the USFR outcome revealed an open-loop structure; therefore, a consistency model was employed for analysis. Results of the standardized mean differences (SMDs) and their 95% confidence intervals showed that, compared to routine care, oral moisturizing gel produced the most significant improvement in unstimulated salivary flow rate in patients with HNC (SMD = 3.83, 95% CI [0.56, 7.09]), with a statistically significant between-group difference. Photobiomodulation therapy also demonstrated superior efficacy over routine care (SMD = 2.41, 95% CI [0.70, 4.11]). In addition, the effects of mouthwash and acupuncture on increasing unstimulated salivary flow rate were found to be comparable (SMD = 0.03, 95% CI [-3.68, 3.62]). Detailed results are presented in **Figure 6**.

**Figure 7:**
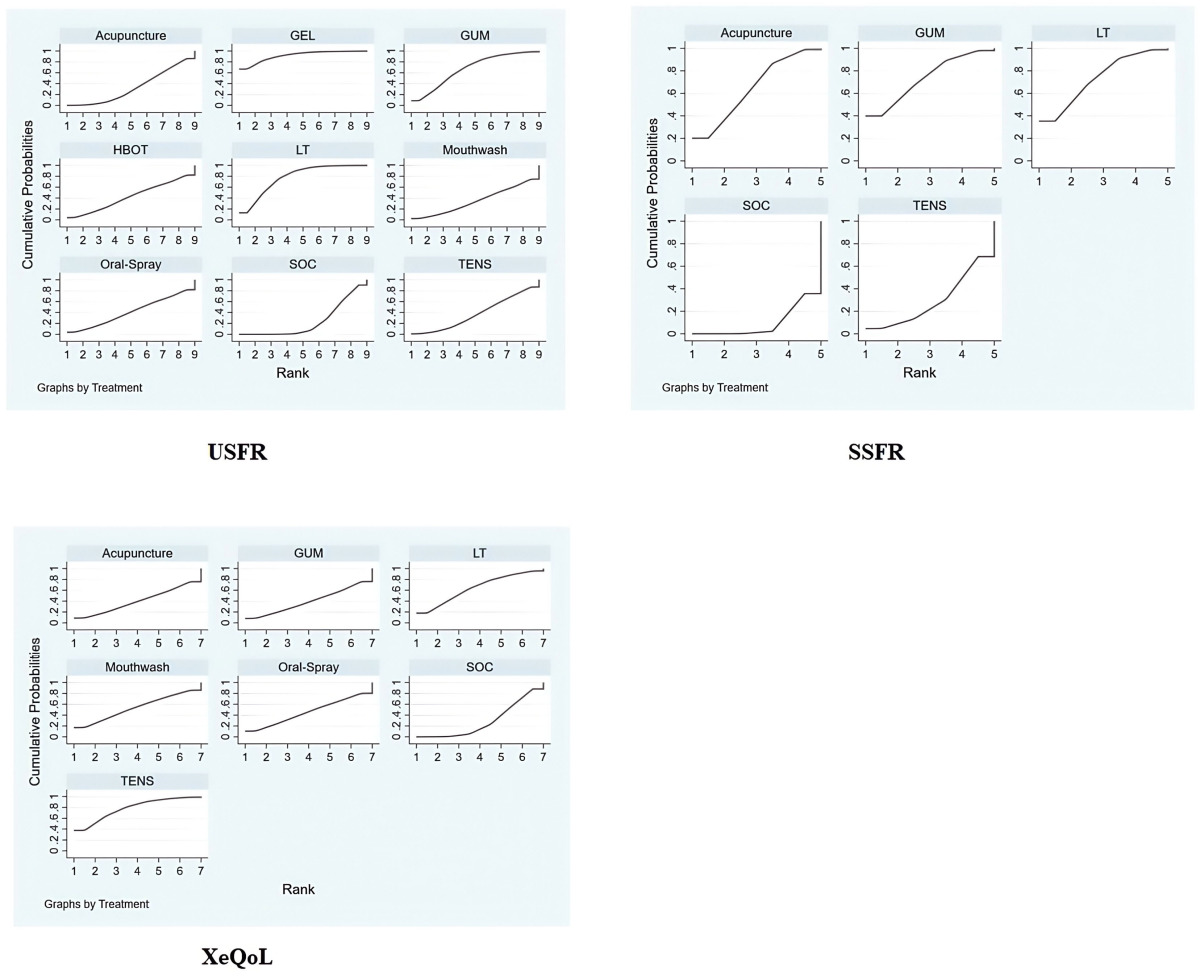
Bayesian ranking plot of non-pharmacological interventions for xerostomia in patients with HNC following radiotherapy, based on efficacy outcomes in USFR, SSFR, and XeQoL scores.

Top-ranked interventions: Oral Gel (SUCRA: 91.3%) > Photobiomodulation (77.9%) > Chewing Gum (67.0%) > HBOT (42.6%) > Oral Spray (41.2%) (**Figure 7**).

##### 3.2.2.2 SSFR

Eleven studies (27–30, 32, 35, 36, 42, 43, 46, 50) measured SSFR. The network included Chewing Gum, Photobiomodulation, Acupuncture, TENS, and SOC (**Figure 5**).

**Figure 8:**
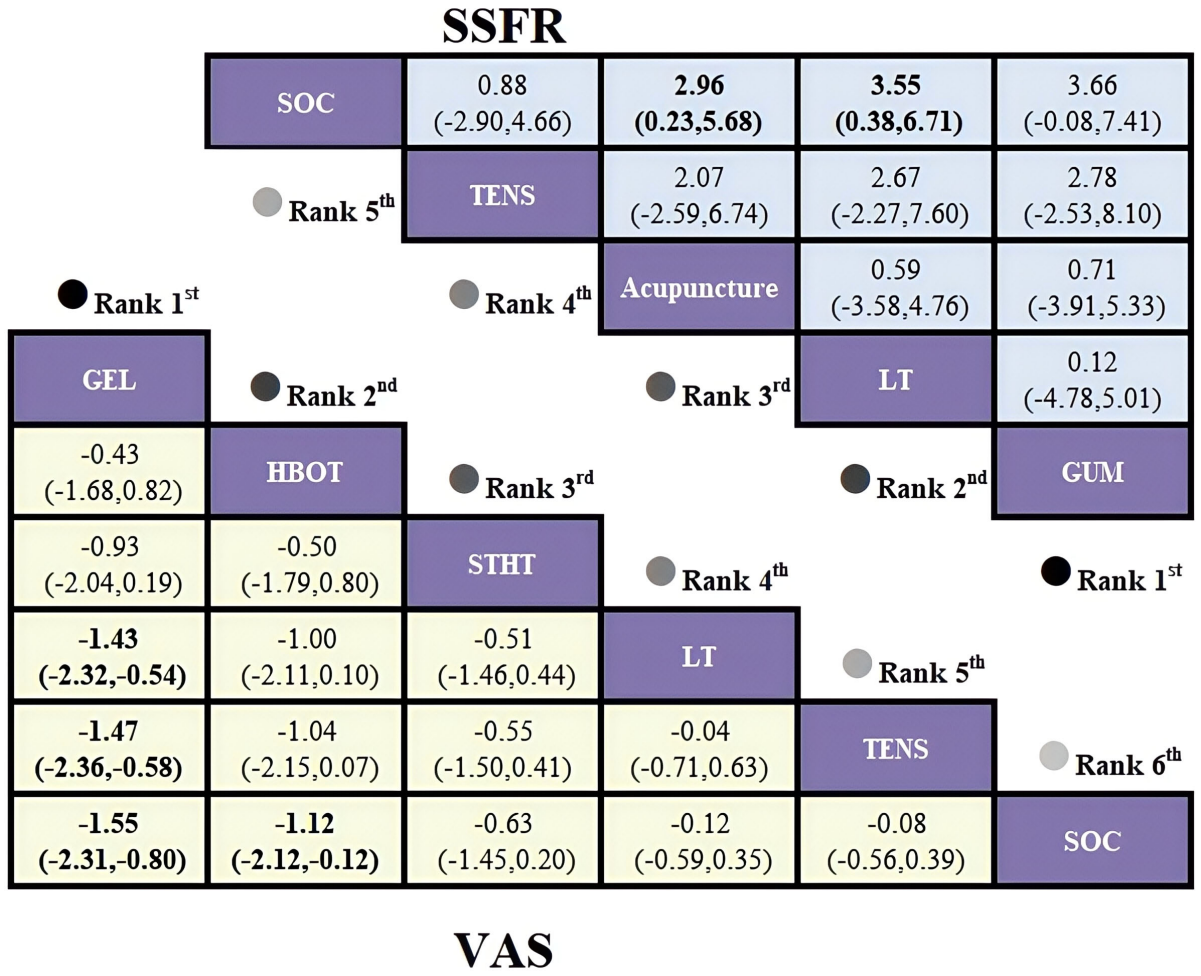
League table based on Bayesian network meta-analysis comparing the efficacy and safety of non-pharmacological interventions for xerostomia in patients with HNC following radiotherapy. A statistically significant difference is indicated when the SMD values and their corresponding 95% confidence intervals for both VAS and SSFR are either entirely above or below zero.

The SSFR-based network revealed an open-loop structure; therefore, a consistency model was applied. Analysis of standardized mean differences (SMDs) and their 95% confidence intervals indicated that chewing gum produced the greatest improvement in stimulated salivary flow rate among HNC patients compared to routine care (SMD = 3.66, 95% CI [-0.08, 7.41]); however, the between-group difference was not statistically significant. In contrast, both photobiomodulation therapy (SMD = 3.55, 95% CI [0.38, 6.71]) and acupuncture (SMD = 2.96, 95% CI [0.23, 5.68]) significantly increased SSFR, with statistically significant differences compared to routine care. Detailed results are presented in **Figure 8**.

SUCRA rankings: chewing gum (74.0%) > photobiomodulation (73.0%) > acupuncture (64.4%) > electrical stimulation (29.3%) > SOC (9.3%) (**Figure 7**).

#### 3.2.3 Secondary Outcome: XeQoL scores

Eight studies (19, 27, 33, 44, 48, 52–54) utilized the XeQoL. The network included seven interventions: Chewing Gum, Photobiomodulation, Acupuncture, TENS, Mouthwash, Oral Spray, and SOC (**Figure 5**).

The network structure based on the XeQoL outcome was open-loop; therefore, a consistency model was applied. Analysis of standardized mean differences (SMDs) and their 95% confidence intervals showed that, compared to routine care, none of the interventions—electrical stimulation therapy (SMD = -1.82, 95% CI [-3.96, 0.33]), photobiomodulation therapy (SMD = -1.21, 95% CI [-3.37, 0.95]), mouthwash (SMD = -0.84, 95% CI [-3.82, 2.15]), oral spray (SMD = -0.48, 95% CI [-3.46, 2.49]), chewing gum (SMD = -0.20, 95% CI [-3.15, 2.75]), or acupuncture (SMD = -0.23, 95% CI [-3.18, 2.73])—produced statistically significant improvements in XeQoL. Detailed results are presented in **Figure 6**.

SUCRA rankings: TENS (78.1%) > Photobiomodulation (64.0%) > Mouthwash (53.1%) > Oral Spray (45.9%) > Chewing Gum (40.3%) (**Figure 7**).

### 3.3 Assessment of publication bias

**Figure 9:**
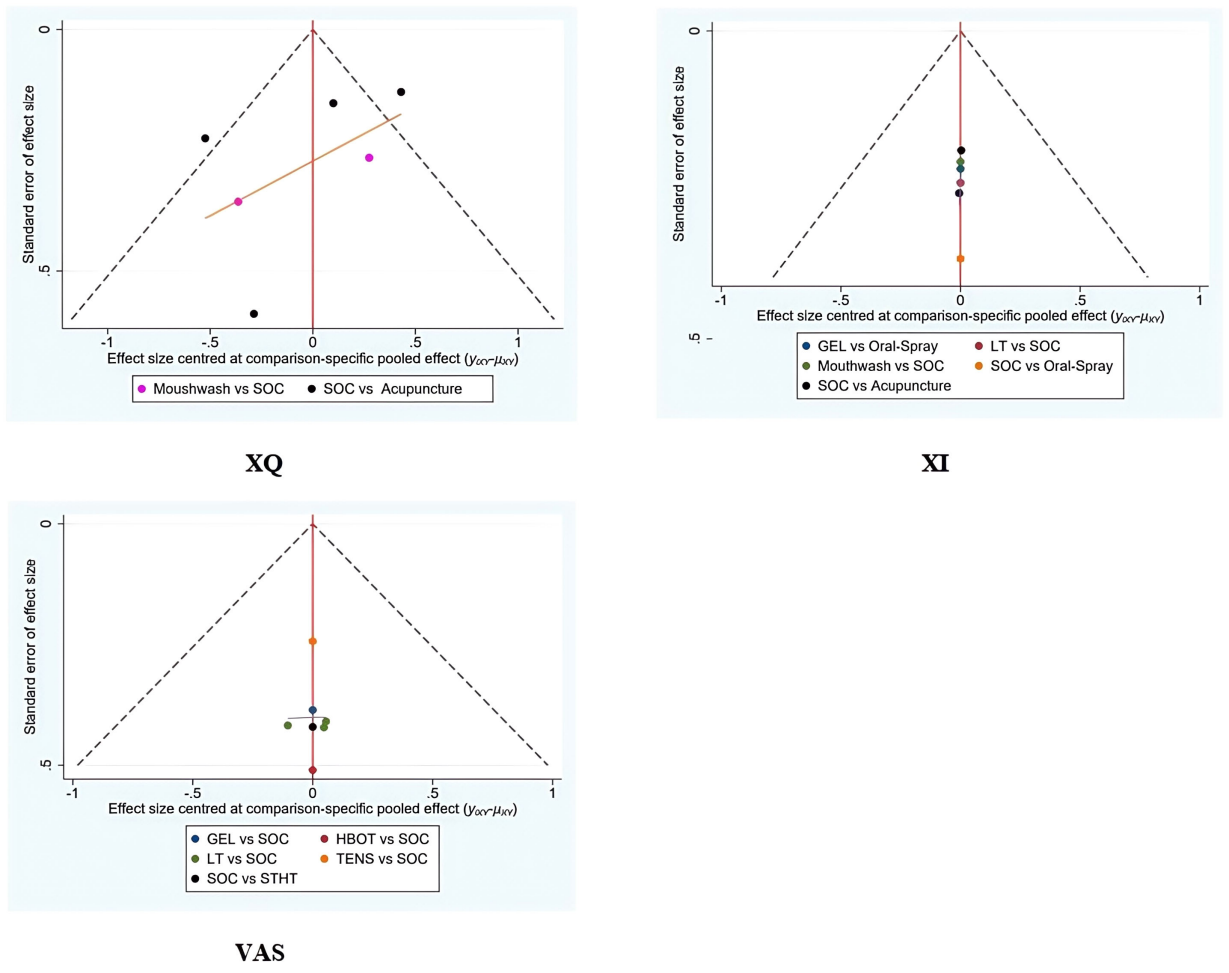
Funnel plots assessing the efficacy and safety of non-pharmacological interventions for xerostomia in patients with HNC following radiotherapy, based on the outcome measures XQ, XI, and VAS.

**Figure 10:**
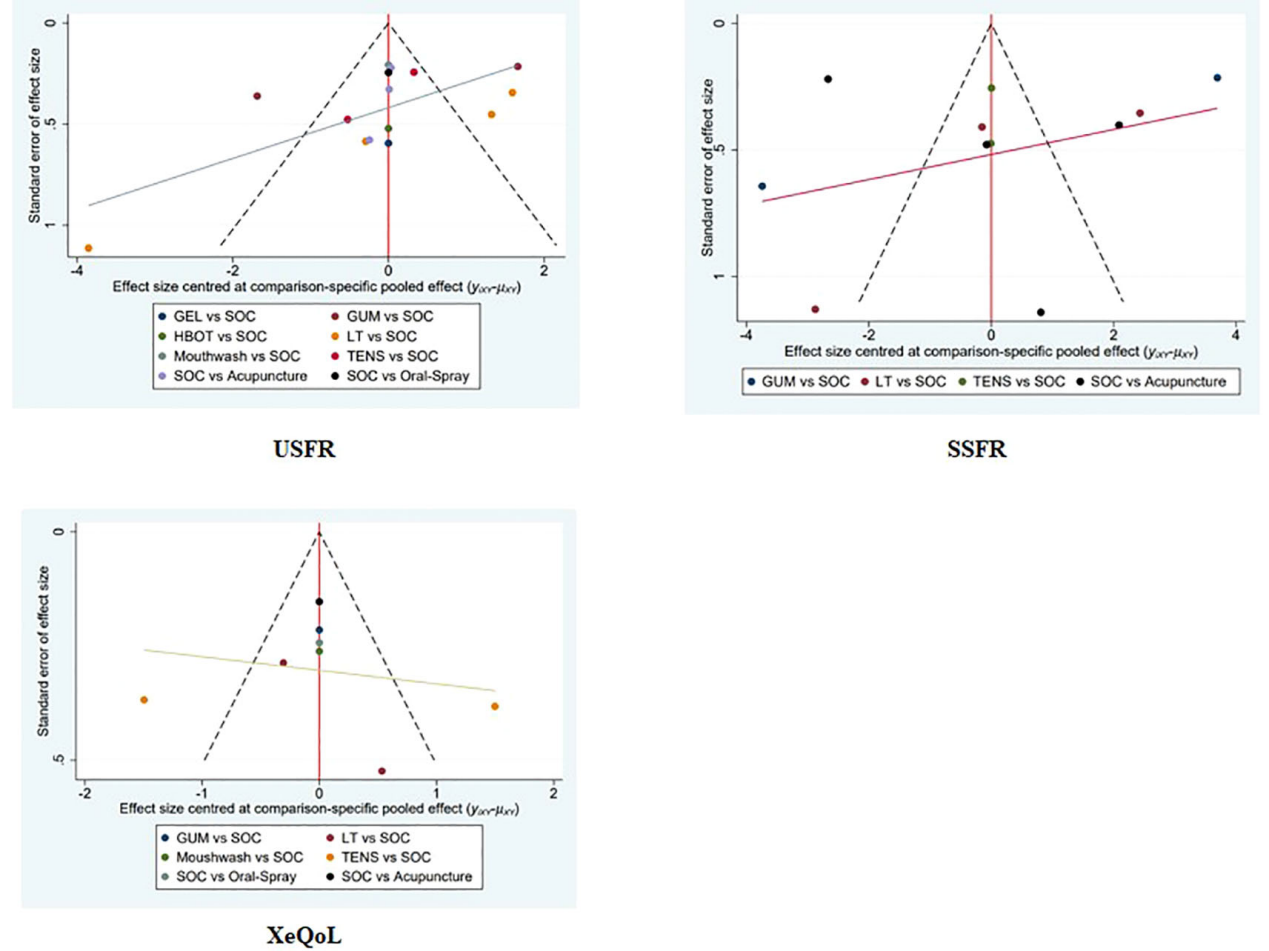
Funnel plots assessing the efficacy and safety of non-pharmacological interventions for xerostomia in patients with HNC following radiotherapy, based on the outcome measures USFR, SSFR, and XeQoL.

Funnel plots were generated for all primary and secondary outcomes (XQ, XI, VAS, USFR, SSFR, XeQoL). The plots showed symmetrical distributions, with no apparent asymmetry or outliers, suggesting a low risk of publication bias (**Figures 9**, **10**).

## 4 Discussion

To the best of our knowledge, this is the first comprehensive systematic review and network meta-analysis to evaluate the safety and efficacy of non-pharmacological interventions for xerostomia in patients with HNC following radiotherapy. The findings of this study offer high-level evidence to inform clinical decision-making, with key conclusions summarized as follows:

(1) Artificial saliva substitutes, including mouthwashes and oral moisturizing gels, demonstrated superior efficacy in alleviating xerostomia symptoms compared with SOC, although no significant safety advantage was observed. Notably, photobiomodulation significantly improved both salivary flow rates and quality of life (QoL) relative to SOC.
(2) Among all interventions, mouthwashes yielded the most favorable outcomes in terms of symptom relief, with a statistically significant benefit over SOC. Oral moisturizing gels also performed well, ranking highly for their effectiveness in mitigating dryness.
(3) Oral moisturizing gels improved unstimulated salivary flow most, with a statistically significant advantage over SOC. Chewing gum emerged as the most effective intervention for stimulating salivary output under gustatory or masticatory conditions, although the difference from SOC was not statistically significant. photobiomodulation demonstrated robust efficacy in enhancing unstimulated and stimulated salivary flow, achieving statistically significant improvements over SOC.
(4) TENS was identified as the most effective strategy for improving xerostomia-related quality of life, outperforming all other evaluated interventions.

Overall, non-pharmacological interventions showed clear advantages over SOC in both symptom relief and salivary enhancement, consistent with previous meta-analyses (56). Distinct from prior studies, our analysis also incorporated long-term outcomes, highlighting that novel therapies such as photobiomodulation and TENS can meaningfully improve patients’ quality of life. Given the heterogeneity in efficacy and safety profiles across interventions, our study presents a stratified evaluation, thereby providing more nuanced and objective conclusions. The underlying mechanism of artificial saliva likely lies in its polymer-based matrices (e.g., carboxymethylcellulose, xanthan gum), which form a protective film on the oral mucosa. This reduces surface tension, maintains mucosal hydration, and minimizes friction between mucosal surfaces, effectively alleviating dryness, cracking, and other discomforts associated with hyposalivation (57, 58). This may explain its superior efficacy over SOC. Chewing gum, on the other hand, enhances salivary flow through the combined stimulation of gustatory and masticatory receptors. This dual mechanism is particularly beneficial for patients with salivary gland hypofunction, consistent with previous findings by Dodds et al. (59–61). Although indirect comparisons in our analysis suggest that artificial saliva substitutes may outperform chewing gum in increasing unstimulated salivary output, the differences were not statistically significant.

Interestingly, emerging therapies such as photobiomodulation and TENS demonstrated significant advantages over SOC in improving patient-reported quality of life. These modalities convert light energy into biochemical activity, promoting tissue repair and modulating inflammatory responses. Additionally, they facilitate neovascularization and collagen synthesis within salivary glands, thereby enhancing glandular function and improving mastication and swallowing abilities (32, 44, 62, 63). Improved oral intake supports nutritional status and oral health, further enhancing QoL. In contrast, mechanical and topical interventions such as chewing gum and artificial saliva primarily directly affect salivary secretion, offering more immediate symptomatic relief, which may explain their superior performance in short-term symptom management relative to SOC.

## 5 Implications

This study represents the most comprehensive synthesis of RCT data to assess the efficacy and safety of non-pharmacological interventions for managing radiation-induced xerostomia. The findings offer clinically relevant evidence to guide therapeutic decision-making. Saliva substitutes, such as mouthwashes and oral moisturizing gels, emerge as favorable first-line options for symptom relief. Specifically, oral moisturizing gels demonstrated significant benefits in increasing unstimulated salivary flow, while chewing gum proved more effective in stimulating salivary secretion through mechanical and gustatory pathways. Notably, TENS was identified as the most promising approach for improving XeQoL. These results can potentially inform future updates to clinical guidelines, including those issued by the American Society of Clinical Oncology (ASCO), regarding best practices for managing xerostomia in patients with HNC post-radiotherapy.

Although the included RCTs involved diverse populations and were conducted across multiple international centers, most participants were recruited from Europe, North America, and Asia. To enhance the generalizability and applicability of these findings, future trials should prioritize the inclusion of populations from underrepresented regions, such as Africa and Indigenous communities. Additionally, the effectiveness of non-pharmacological therapies is closely linked to patient adherence. Future research should explore behavioral, cultural, and logistical factors that influence compliance, as understanding these determinants will be instrumental in optimizing real-world treatment outcomes for patients with xerostomia.

## 6 Limitations

Despite the strengths of this analysis, several limitations should be acknowledged. First, heterogeneity in the definition and implementation of SOC across control groups may have introduced inconsistencies in comparative outcomes. For example, sham acupuncture was used as a control in some trials, whereas placebo oral treatments were employed in others—both considered forms of SOC, yet differing in their contextual and psychological impact. Second, none of the included studies enrolled participants of African descent, limiting the applicability of the findings to Black populations and other ethnically underrepresented groups. This highlights the urgent need for more inclusive research to ensure equity in evidence-based care. Third, variability in the duration, intensity, and frequency of non-pharmacological interventions across trials may have influenced effect sizes and introduced clinical heterogeneity. These differences complicate direct comparisons and may affect the generalizability of specific treatment protocols. Despite these limitations, this meta-analysis offers a robust and systematic evaluation of non-pharmacological therapies for radiation-induced xerostomia in HNC patients, delivering valuable insights for future research and clinical practice. The stratified evaluation of interventions presented here supports tailored, evidence-informed treatment strategies to improve patient outcomes.

## Data Availability

The original contributions presented in the study are included in the article/**Supplementary Material**. Further inquiries can be directed to the corresponding author.
