# Supplementary material for: Comparative efficacy and safety of non-pharmacological interventions on treatment-induced xerostomia in head and neck cancer patients: a systematic review and network meta-analysis
Source: Front Oncol. 2025 Jul 30;15:1644178. doi: 10.3389/fonc.2025.1644178 (PMC12343224; doi:10.3389/fonc.2025.1644178)
Supplement: Supplementary file 2 [file Image1.pdf]

**Table S2.Literature Search Strategy**

| | |
|---|---|
| **Pubmed** | #1 (((((((((Xerostomia[MeSH Terms]) OR (Xerostomias[Title/Abstract])) OR (Asialia[Title/Abstract])) OR (Asialias[Title/Abstract])) OR ("Mouth Dryness"[Title/Abstract])) OR ("Dryness, Mouth"[Title/Abstract])) OR (Hyposalivation[Title/Abstract])) OR (Hyposalivations[Title/Abstract])) OR (Thirst[Title/Abstract])) OR (Thirsts[Title/Abstract]) 26005<br>#2 ((((“head and neck neoplasms”[MeSH Terms]) ) OR (“Pharyngeal Neoplasms”[Title/Abstract])) OR (“squamous cell carcinoma of head and neck”[Title/Abstract])) OR (“Cancer of Head and Neck”[Title/Abstract]) 369,276<br>#3 (((((((((((((((((((((((((((((((((((((((((((((((((((((((((((((((((((((((((((((((((ice[MeSH Terms]) OR ("common cold"[Title/Abstract])) OR (“cold temperature”[Title/Abstract])) OR (cool[Title/Abstract])) OR (popsicle[Title/Abstract])) OR ("Low-Level Light Therapy"[MeSH Terms])) OR ("Light Therapies, Low-Level"[Title/Abstract])) OR ("Light Therapy, Low-Level"[Title/Abstract])) OR ("Low-Level Light Therapies"[Title/Abstract])) OR ("Low Level Light Therapy"[Title/Abstract])) OR ("Therapies, Low-Level Light"[Title/Abstract])) OR ("Therapy, Low-Level Light"[Title/Abstract])) OR (LLLT[Title/Abstract])) OR ("Photobiomodulation Therapy"[Title/Abstract])) OR ("Photobiomodulation Therapies"[Title/Abstract])) OR ("Therapies, Photobiomodulation"[Title/Abstract])) OR ("Therapy, Photobiomodulation"[Title/Abstract])) OR (Photobiomodulation[Title/Abstract])) OR (Photobiomodulations[Title/Abstract])) OR ("Laser Therapy, Low-Level"[Title/Abstract])) OR ("Laser Therapies, Low-Level"[Title/Abstract])) OR ("Laser Therapy, Low Level"[Title/Abstract])) OR ("Low-Level Laser Therapies"[Title/Abstract])) OR ("Laser Biostimulation"[Title/Abstract])) OR ("Biostimulation, Laser"[Title/Abstract])) OR ("Laser Irradiation, Low-Power"[Title/Abstract])) OR ("Irradiation, Low-Power Laser"[Title/Abstract])) OR ("Laser Irradiation, Low Power"[Title/Abstract])) OR ("Laser Phototherapy"[Title/Abstract])) OR ("Phototherapy, Laser"[Title/Abstract])) OR ("Laser Therapy, Low-Power"[Title/Abstract])) OR ("Laser Therapies, Low-Power"[Title/Abstract])) OR ("Laser Therapy, Low Power"[Title/Abstract])) OR ("Low-Power Laser Therapies"[Title/Abstract])) OR ("Low-Level Laser Therapy"[Title/Abstract])) OR ("Low Level Laser Therapy"[Title/Abstract])) OR ("Low-Power Laser Irradiation"[Title/Abstract])) OR ("Low Power Laser Irradiation"[Title/Abstract])) OR ("Low-Power Laser Therapy"[Title/Abstract])) OR ("Low Power Laser Therapy"[Title/Abstract])) OR ("Chewing Gum"[MeSH Terms])) OR ("Chewing Gums"[Title/Abstract])) OR ("Gum, Chewing"[Title/Abstract])) OR ("Gums, Chewing"[Title/Abstract])) OR (Menthol[MeSH Terms]))) OR (Cyclohexanol[Title/Abstract])) OR (Acupuncture[MeSH Terms])) OR (Pharmacopuncture[Title/Abstract])) OR ("nerve stimulation"[MeSH Terms])) OR ("Electric Stimulation"[Title/Abstract])) OR ("Stimulation, Electric"[Title/Abstract])) OR ("Electric Stimulations"[Title/Abstract])) OR ("Stimulations, Electric"[Title/Abstract])) OR ("Electrical Stimulation"[Title/Abstract])) OR ("Electrical Stimulations"[Title/Abstract])) OR ("Stimulation, Electrical"[Title/Abstract])) OR ("Stimulations, Electrical"[Title/Abstract])) OR ("ear acupressure"[MeSH Terms])) OR ("auricular pressure"[Title/Abstract])) OR ("auricular point sticking"[Title/Abstract])) OR ("auricular-plaster therapy"[Title/Abstract])) OR (auriculotherapy[Title/Abstract]))) OR ("Psychological intervention"[MeSH Terms])) OR ("Intervention, Psychosocial"[Title/Abstract])) OR ("Interventions, Psychosocial"[Title/Abstract])) OR ("Psychosocial Interventions"[Title/Abstract])) OR ("Psychological Intervention"[Title/Abstract])) OR ("Intervention, Psychological"[Title/Abstract])) OR ("Interventions, Psychological"[Title/Abstract])) OR ("Psychological Interventions"[Title/Abstract]) 151757<br>#4 ((randomized controlled trial[Publication Type]) OR (randomized[Title/Abstract])) OR (placebo[Title/Abstract]) 1098275<br>#5 #1 AND#2 AND#3 AND#4 194 |
| **Web of Science** | 1: (((((((((TS=(Xerostomia)) OR TS=(Xerostomias)) OR TS=(Asialia)) OR TS=(Asialias)) OR TS=("Mouth Dryness")) OR TS=("Dryness, Mouth")) OR TS=(Hyposalivation)) OR TS=(Hyposalivations)) OR TS=(Thirst)) OR TS=(Thirsts) 28254<br>2： (((TS=(“head and neck neoplasms”)) OR TS=(“Pharyngeal Neoplasms”)) OR TS=(“squamous cell carcinoma of head and neck”)) OR TS=(“Cancer of Head and Neck”) 80,585<br>3:(((((((((((((((((((((((((((((((((((((((((((((((((((((((((((((((((((((((((((((((((TS=(ice)) OR TS=(common cold)) OR TS=(“cold temperature”)) OR TS=(cool)) OR TS=(popsicle)) OR TS=("Low-Level Light Therapy")) OR TS=("Light Therapies, Low-Level")) OR TS=("Light Therapy, Low-Level")) OR TS=("Low-Level Light Therapies")) OR TS=("Low Level Light Therapy")) OR TS=("Therapies, Low-Level Light")) OR TS=("Therapy, Low-Level Light")) OR TS=(LLLT)) OR TS=("Photobiomodulation Therapy")) OR TS=("Photobiomodulation Therapies")) OR TS=("Therapies, Photobiomodulation")) OR TS=("Therapy, Photobiomodulation")) OR TS=(Photobiomodulation)) OR TS=(Photobiomodulations)) OR TS=("Laser Therapy, Low-Level")) OR TS=("Laser Therapies, Low-Level")) OR TS=("Laser Therapy, Low Level")) OR TS=("Low-Level Laser Therapies")) OR TS=("Laser Biostimulation")) OR TS=("Biostimulation, Laser")) OR TS=("Laser Irradiation, Low-Power")) OR TS=("Irradiation, Low-Power Laser")) OR TS=("Laser Irradiation, Low Power")) OR TS=("Laser Phototherapy")) OR TS=("Phototherapy, Laser")) OR TS=("Laser Therapy, Low-Power")) OR TS=("Laser Therapies, Low-Power")) OR TS=("Laser Therapy, Low Power")) OR TS=("Low-Power Laser Therapies")) OR TS=("Low-Level Laser Therapy")) OR TS=("Low Level Laser Therapy")) OR TS=("Low-Power Laser Irradiation")) OR TS=("Low Power Laser Irradiation")) OR TS=("Low-Power Laser Therapy")) OR TS=("Low Power Laser Therapy")) OR TS=("Chewing Gum")) OR TS=("Chewing Gums")) OR TS=("Gum, Chewing")) OR TS=("Gums, Chewing")) OR TS=(Menthol)) OR TS=(Cyclohexanol)) OR TS=(Acupuncture)) OR TS=(Pharmacopuncture)) OR TS=("nerve stimulation")) OR TS=("Electric Stimulation")) OR TS=("Stimulation, Electric")) OR TS=("Electric Stimulations")) OR TS=("Stimulations, Electric")) OR TS=("Electrical Stimulation")) OR TS=("Electrical Stimulations")) OR TS=("Stimulation, Electrical")) OR TS=("Stimulations, Electrical")) OR TS=("ear acupressure")) OR TS=("auricular pressure")) OR TS=("auricular point sticking")) OR TS=("auricular-plaster therapy")) OR TS=(auriculotherapy)) OR TS=("Psychological intervention")) OR TS=("Intervention, Psychosocial")) OR TS=("Interventions, Psychosocial")) OR TS=("Psychosocial Interventions")) OR TS=("Psychological Intervention")) OR TS=("Intervention, Psychological")) OR TS=("Interventions, Psychological")) OR TS=("Psychological Interventions") 4,195,052<br>4: ((TS=("randomized controlled trial")) OR TS=(randomized)) OR TS=(placebo) 1,474,269<br>5: #1 AND #2 AND #3 AND #4 294 |
| **Cochrane** | #1 MeSH descriptor: [Xerostomia] explode all trees1239<br>#2 (Xerostomias OR Asialia OR Asialias OR "Mouth Dryness" OR "Dryness, Mouth" OR Hyposalivation OR Hyposalivations OR Thirst OR Thirsts):ti,ab,kw (Word variations have been searched) 7161<br>#3 #1 OR #2 7517<br>#4 MeSH descriptor: [Ice] explode all trees 217<br>#5 (common cold OR “cold temperature” OR cool OR popsicle):ti,ab,kw (Word variations have been searched) 10936 |

| | |
|---|---|
| | #6 MeSH descriptor: [Low-Level Light Therapy] explode all trees 1730<br>#7 ('light therapies, low-level' OR 'light therapy, low-level' OR 'low-level light therapies' OR 'low level light therapy' OR 'therapies, low-level light' OR 'therapy, low-level light' OR lllt OR 'photobiomodulation therapy' OR 'photobiomodulation therapies' OR 'therapies, photobiomodulation' OR 'therapy, photobiomodulation' OR photobiomodulation OR photobiomodulations OR 'laser therapy, low-level' OR 'laser therapies, low-level' OR 'laser therapy, low level' OR 'low-level laser therapies' OR 'laser biostimulation' OR 'biostimulation, laser' OR 'laser irradiation, low-power' OR 'irradiation, low-power laser' OR 'laser irradiation, low power' OR 'laser phototherapy' OR 'phototherapy, laser' OR 'laser therapy, low-power' OR 'laser therapies, low-power' OR 'laser therapy, low power' OR 'low-power laser therapies' OR 'low-level laser therapy' OR 'low level laser therapy' OR 'low-power laser irradiation' OR 'low power laser irradiation' OR 'low-power laser therapy' OR 'low power laser therapy'):ti,ab,kw (Word variations have been searched) 6683<br>#8 MeSH descriptor: [Chewing Gum] explode all trees 845<br>#9 ('Chewing Gums' OR 'Gum, Chewing' OR 'Gums, Chewing'):ti,ab,kw (Word variations have been searched) 2031<br>#10 MeSH descriptor: [Menthol] explode all trees 438<br>#11 MeSH descriptor: [Acupuncture] explode all trees 225<br>#12 (Pharmacopuncture):ti,ab,kw (Word variations have been searched) 113<br>#13 MeSH descriptor: [Electric Stimulation] explode all trees 2632<br>#14 ('nerve stimulation' OR 'stimulation, electric' OR 'electric stimulations' OR 'stimulations, electric' OR 'electrical stimulation' OR 'electrical stimulations' OR 'stimulation, electrical' OR 'stimulations, electrical'):ti,ab,kw (Word variations have been searched) 25087<br>#15 ('ear acupressure' OR 'auricular acupressure' OR 'auricular acupressure' OR 'auricular pressure' OR 'auricular point sticking' OR 'auricular-plaster therapy' OR 'auriculotherapy' OR 'auriculotherapy'):ti,ab,kw (Word variations have been searched) 1236<br>#16 MeSH descriptor: [Psychosocial Intervention] explode all trees 313<br>#17 ('intervention, psychosocial' OR 'interventions, psychosocial' OR 'psychosocial interventions' OR 'psychological intervention OR 'psychological intervention' OR 'intervention, psychological' OR 'interventions, psychological' OR 'psychological interventions'):ti,ab,kw (Word variations have been searched) 76796<br>#18 MeSH descriptor: [head and neck neoplasms] explode all trees 35<br>#19 ('head and neck neoplasms' OR 'Pharyngeal Neoplasms' OR 'squamous cell carcinoma of head and neck' OR 'Cancer of Head and Neck'):ti,ab,kw (Word variations have been searched) 9680<br>#20 #18 OR #19<br>#21 #4 OR #5 OR #6 OR #7 OR #8 OR #9 OR #10 OR #11 OR #12 OR #13 OR #14 OR #15 OR #16 OR #17 121835<br>#22 #3 AND #20 AND #21 160 |
| **Embase** | #33 #11 AND #26 AND #29 AND #32 619<br>#32 #30 OR #31 23,031<br>#31 'head and neck neoplasms' OR 'pharyngeal neoplasms' OR 'squamous cell carcinoma of head and neck' 4,271<br>#30 'head and neck cancer'/exp 364,356<br>#29 #27 OR #28 1,802,686<br>#28 randomized OR placebo 1,801,138<br>#27 'randomized controlled trial'/exp OR 'randomized controlled trial' 1,161,232<br>#26 #12 OR #13 OR #14 OR #15 OR #16 OR #17 OR #18 OR #19 OR #20 OR #21 OR #22 OR #23 OR #24 OR #25 479,386<br>#25 'intervention, psychosocial' OR 'interventions, psychosocial' OR 'psychosocial interventions' OR 'psychological intervention'/exp OR 'psychological intervention' OR 'intervention, psychological' OR 'interventions, psychological' OR 'psychological interventions' 22,305<br>#24 'ear acupressure' OR 'auricular acupressure'/exp OR 'auricular acupressure' OR 'auricular pressure' OR 'auricular point sticking' OR 'auricular-plaster therapy' OR 'auriculotherapy'/exp OR 'auriculotherapy' 1,709<br>#23 'electric stimulation' OR 'stimulation, electric' OR 'electric stimulations' OR 'stimulations, electric' OR 'electrical stimulation' OR 'electrical stimulations' OR 'stimulation, electrical' OR 'stimulations, electrical' 75,694<br>#22 'nerve stimulation'/exp OR 'nerve stimulation' 205,311<br>#21 pharmacopuncture:ab,ti 383<br>#20 'acupuncture'/exp OR 'acupuncture' 75,227<br>#19 cyclohexanol:ab,ti1 251<br>#18 'menthol'/exp OR 'menthol' 8,444<br>#17 'chewing gum'/exp OR 'chewing gum' 4,152<br>#16 'chewing gums' OR 'gum, chewing' OR 'gums, chewing' 1,104<br>#15 'light therapies, low-level' OR 'light therapy, low-level' OR 'low-level light therapies' OR 'low level light therapy' OR 'therapies, low-level light' OR 'therapy, low-level light' OR lllt OR 'photobiomodulation therapy' OR 'photobiomodulation therapies' OR 'therapies, photobiomodulation' OR 'therapy, photobiomodulation' OR photobiomodulation OR photobiomodulations OR 'laser therapy, low-level' OR 'laser therapies, low-level' OR 'laser therapy, low level' OR 'low-level laser therapies' OR 'laser biostimulation' OR 'biostimulation, laser' OR 'laser irradiation, low-power' OR 'irradiation, low-power laser' OR 'laser irradiation, low power' OR 'laser phototherapy' OR 'phototherapy, laser' OR 'laser therapy, low-power' OR 'laser therapies, low-power' OR 'laser therapy, low power' OR 'low-power laser therapies' OR 'low-level laser therapy' OR 'low level laser therapy' OR 'low-power laser irradiation' OR 'low power laser irradiation' OR 'low-power laser therapy' OR 'low power laser therapy' 31,124<br>#14 'low level laser therapy'/exp OR 'low level laser therapy' 28,830<br>#13 'common cold' OR 'cold temperature' OR cool OR popsicle 35,971<br>#12 'ice'/exp OR 'ice' 62,710<br>#11 #1 OR #2 OR #3 OR #4 OR #5 OR #6 OR #7 OR #8 OR #9 OR #10 56391<br>#10 thirsts:ab,ti 18<br>#9 thirst:ab,ti 6,525<br>#8 hyposalivations:ab,ti 0<br>#7 hyposalivation:ab,ti 1,104<br>#6 'dryness, mouth':ab,ti 8<br>#5 'mouth dryness':ab,ti 490<br>#4 asialias:ab,ti 0<br>#3 asialia:ab,ti 15<br>#2 xerostomias:ab,ti 8 |

| | |
|---|---|
| #1 'xerostomia'/exp OR 'xerostomia' | 49,703 |
