# Supplementary material for: Comparative efficacy and safety of non-pharmacological interventions on treatment-induced xerostomia in head and neck cancer patients: a systematic review and network meta-analysis
Source: Front Oncol. 2025 Jul 30;15:1644178. doi: 10.3389/fonc.2025.1644178 (PMC12343224; doi:10.3389/fonc.2025.1644178)
Supplement: Supplementary file 3 [file Image2.pdf]

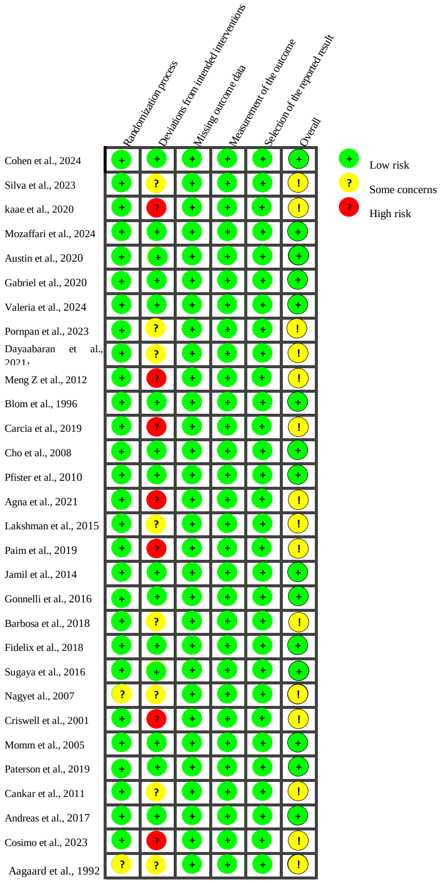
Randomization process
Deviations from intended interventions
Missing outcome data
Measurement of the outcome
Selection of the reported result
Overall
Cohen et al., 2024
Silva et al., 2023
kaae et al., 2020
Mozaffari et al., 2024
Austin et al., 2020
Gabriel et al., 2020
Valeria et al., 2024
Pornpan et al., 2023
Dayaabaran et al., 2021
Meng Z et al., 2012
Blom et al., 1996
Carcia et al., 2019
Cho et al., 2008
Pfister et al., 2010
Agna et al., 2021
Lakshman et al., 2015
Paim et al., 2019
Jamil et al., 2014
Gonnelli et al., 2016
Barbosa et al., 2018
Fidelix et al., 2018
Sugaya et al., 2016
Nagyet al., 2007
Criswell et al., 2001
Momm et al., 2005
Paterson et al., 2019
Cankar et al., 2011
Andreas et al., 2017
Cosimo et al., 2023
Aagaard et al., 1992
Low risk
Some concerns
High risk
